# Abdomino-Perineal Procto-Sigmoidectomy

**Published:** 1899-08

**Authors:** James N. Ellis

**Affiliations:** Atlanta, Ga.


### ABDOMINO-PERINEAL PROCTO-SIGMOIDECTOMY.

By JAMES N. ELLIS, M.D.,

ATLANTA, GA.

Cancer is a disease which is common in its occurrence, increasing in frequency, and unrelenting in its demands for the life of its victim. However caused and disseminated, this disease is primarily local, and while so, is susceptible of cure by radical operation. This being admittedly true, in no field of surgery is the importance of early diagnosis more imperative than in cancer. We cannot prevent its occurrence, as we do not know its cause; but we do know that a sufficiently thorough, early operation will stay its progress and frequently avert its return.

Since the introduction of Kelly's tubes, and their modification by Tuttle, so as to facilitate their introduction above the brim of the pelvis and to permit of visual inspection of the whole of the rectum and sigmoid, the early diagnosis of cancer of the large intestine is rendered possible in positions entirely beyond the reach of such surgical procedures as ordinary perineal or sacral proctectomy. Such operations are of limited application, permitting only of the removal of 15 or 16 centimeters of intestine, and as it is always necessary to go well above the neoplasm, the cancer must lie very near the anus for the operation to be radical. Kraske's method somewhat enlarged the field of this operation, but we may consider that when the disease extends or is situated more than 12 centimeters above the anus some other procedure must be adopted. The inguinal operation contemplates only the removal of a segment of the sigmoid or upper rectum, and, in view of our present knowledge of the infectious nature of cancerous material and its method of dissemination, cannot be considered radical. The probability of incipient foci below, infected by necrotic débris from the primary growth above, is too evident to be ignored, even when such foci are not visually demonstrable.

The surgical procedure which I shall suggest for the removal of these malignant growths is a combination of the inguinal and perineal operations, necessitating the opening of the abdominal cavity, the ligation of the internal iliac arteries, and the making of an artificial anus in the iliac region. It is a grave operation, but if it gives the only, or the best, chance of radically extirpating the neoplasm, neither the patient nor the surgeon will hesitate to resort to it. If our only means of successfully combating this dread, death-dealing disease is a sufficiently early radical operation, any method which facilitates the accomplishment of this desired result will not be rejected by the competent surgeon because of the increased gravity of the operation.

The making of an artificial anus at any point is, at best, a very disagreeable necessity, involving the destruction of the sphincter ani (except in the limited number of cases in which Hochenegg's method is applicable), whether it be in the perineal, high sacral or iliac regions; but experience proves that its reference to the iliac region is not without its advantages, since here it offers less liability to subsequent prolapse and extrusion, than when situated below.

The objection of increased gravity incident to opening the abdomen, which will be urged against the method I am about to suggest, carries but little weight, when the operation is performed by one versed in modern surgical technique. It is only necessary to practice rigorous asepsy during and after the operation, and to avoid unnecessary hemorrhage. This last is best accomplished, as I have intimated, by preliminary ligation of the internal iliac arteries.

Considering these indications, as outlined above, the operation, which contemplates the complete removal of the rectum, and a contiguous portion of the sigmoid, can, briefly, be best performed as follows:

With the operator on the left of the patient, open the abdomen by the usual suprapubic incision. Locate the inferior border of the body of the last lumbar vertebra, at which level the internal iliacs can be felt pulsating, from 3 to 3½ centimeters to each side of the median line. Incise the peritoneal covering, being careful to avoid wounding the ureter, which passes in front of the vessel near its origin, and which is now drawn outward with the outer lip of the incised peritoneum. Ligate the vessel, remembering its intimate relationship with the underlying vein, and repeat the operation on the artery of the opposite side. Liberate the sigmoid flexure of the colon, ligating the inferior mesenteric artery just above the sigmoidal branches, when necessary; pass a mesh of sterilized gauze beneath the gut through the meso-sigmoid, milk away the contents of the gut, tie the gauze around it above, and a ligature several centimeters below, and cut the intestine between the two with a thermocautery, or with scissors, after compression by the ingenious, progressive pressure-clamp of Doyen. Bind a cuff or sterilized rubber cloth or gauze over the distal end, invert and close the proximal end, and, taking up the inferior segment of intestine, continue the liberation of the rectum as far downward as is conveniently possible. To do this, make an incision along the visceral margin of the meso-rectum, separate the rectum from the anterior surface of the sacrum with the finger, drop it into the pelvic basin, and pack above with sterilized compresses. Now close the abdominal incision, and proceed in the usual way to make an artificial anus in the left iliac region. This completes the abdominal part of the operation.

Changing the patient to the lithotomy position, plug the anus firmly, and proceed with the perineal part of the operation. Make an elliptical incision through the skin around the anus, cut the levators, freeing the anterior surface of the rectum from the vesical triangle with care, avoiding the prostate and seminal vesicles in the male and the uterus in the female, and, continuing up to the point of the previous downward dissection from the abdomen, complete the operation by removing the whole of the severed intestine, with the compresses above, somewhat after the manner of delivering the uterus in vaginal hysterectomy.

The after-treatment contemplates occlusion by granulation. The cavity is packed with gauze and permitted to close from above downwards.

This operation may be extended to include the uterus or bladder when either of these organs is also involved.

I claim no special originality for the procedure above described, having simply attempted to combine the best points observed in somewhat similar operations performed by Doyen, Gussenbauer, Hochenegg, Quenu, Allingham and von Bergmann. It permits of radically removing the malignant growth, and, although necessarily attended by a considerable mortality in the hands of inexperienced surgeons, it is easy of execution by one versed in abdominal surgery, and causes much less shock than would be supposed.

*19 Forest Avenue.*

